# Dual Modulation of Cardiac Ion Pumps: A Small-Molecule SERCA2a SUMOylation Enhancer Also Inhibits the Na^+^/K^+^-ATPase

**DOI:** 10.3390/biomedicines13123036

**Published:** 2025-12-10

**Authors:** Carlos Cruz-Cortés, Jaroslava Šeflová, L. Michel Espinoza-Fonseca

**Affiliations:** 1 Division of Cardiovascular Medicine, Department of Internal Medicine, University of Michigan, Ann Arbor, MI 48109, USA; ccruzcor@med.umich.edu; 2 Center for Arrhythmia Research, University of Michigan, Ann Arbor, MI 48109, USA; 3 Department of Cell and Molecular Physiology, Loyola University Chicago, Maywood, IL 60153, USA; jseflova@luc.edu

**Keywords:** Na^+^/K^+^-ATPase, inhibitor, dual effectors, SERCA2a, cardiac contractility, inotropic modulation

## Abstract

**Background**: The Na^+^/K^+^-ATPase (NKA) maintains electrochemical gradients by exporting Na+ and importing K^+^ at the expense of ATP hydrolysis. Although NKA inhibition is a well-established strategy for increasing cardiac contractility, existing inhibitors such as cardiotonic steroids (CTS) are limited by serious adverse effects. N106 is a small molecule previously shown to enhance cardiac lusitropy by promoting SERCA2a SUMOylation and, intriguingly, also exerts positive inotropic effects, suggesting additional mechanisms of action. **Methods**: To test whether N106 directly modulates NKA, we combined ATPase activity assays with molecular docking and microsecond-scale molecular dynamics simulations. **Results**: Biochemical measurements showed that N106 partially inhibits NKA, achieving ~80% maximal inhibition with an IC50 of 7 ± 1 µM, while leaving the pump’s apparent affinity for Na^+^, K^+^, and ATP unchanged. Computational analyses suggest that N106 binds within the canonical CTS-binding pocket but undergoes intermittent unbinding events, consistent with the partial inhibition observed experimentally. **Conclusions**: These findings identify N106 as a first-in-class dual modulator of cardiac ion pumps, partially inhibiting NKA while previously shown to activate SERCA2a through enhanced SUMOylation. This combined mechanism likely underlies its positive inotropic and lusitropic effects and positions the N106 scaffold as a promising lead for developing next-generation dual-target therapeutics for heart failure.

## 1. Introduction

Heart failure (HF) remains the leading cause of morbidity and mortality in the United States, accounting for approximately one in nine deaths and an annual economic burden exceeding $32 billion [CDC]. Despite substantial advances in pharmacologic and device-based therapies, HF continues to carry a poor prognosis, with a 5-year mortality rate of roughly 50%. Current treatment strategies primarily target peripheral symptoms, e.g., hypertension or volume overload, but they do not directly address the molecular mechanisms that underlie cardiac dysfunction [1,2]. Even under optimized clinical management, patient outcomes remain suboptimal, underscoring the urgent need for targeted therapies that restore cardiomyocyte function rather than compensating for peripheral symptoms [3,4].

Among the molecular pathways implicated in HF pathogenesis, dysregulation of calcium handling plays a central role in impaired cardiac function. In particular, the cardiac sarcoplasmic reticulum Ca^2+^-ATPase (SERCA2a) is essential for Ca^2+^ reuptake into the sarcoplasmic reticulum during diastole, and its downregulation and dysregulation is a hallmark of HF. Therefore, restoring SERCA2a activity has therefore emerged as a promising target to improve cardiac function. Recent work has identified post-translational SUMOylation of SERCA2a as a key regulator of its stability and catalytic efficiency [5]. SUMOylation is a reversible post-translational modification in which small ubiquitin-like modifier (SUMO) proteins are covalently attached to SERCA2a residues K480 and K585 [5,6]. Enhancing SERCA2a SUMOylation restores calcium cycling and contractile function [5], providing a promising therapeutic strategy for HF [6]. Based on these studies, Kho et al. identified N106, a small molecule that stimulates the catalytic cycle of the ubiquitin-conjugating enzyme 9, thus promoting SERCA2a SUMOylation [7]. As a result, SUMOylation increases SERCA2a activity, stability, and overall function, thus explaining the positive lusitropy observed in previous studies [7].

Interestingly, N106 also exhibits positive inotropic effects [7], suggesting that its pharmacological effects extend beyond modulation of SERCA2a SUMOylation and likely involve other ion transport systems that contribute to excitation–contraction coupling. Among these, inhibition of the Na^+^/K^+^-ATPase (NKA) is a plausible mechanism explaining the positive inotropic effects of N106 because of the physiological link between Na^+^ and Ca^2+^ gradients via the sodium-calcium exchanger, which uses the electrochemical gradient of Na^+^ ions to extrude Ca^2+^ from cells [8]. Therefore, in this study we used molecular docking, ATPase assays, and molecular dynamics (MD) simulations to investigate whether N106 directly acts as an effector of NKA. Here we show that N106 inhibits NKA at concentrations previously shown to enhance SERCA2a SUMOylation [7]. Molecular docking and microsecond-long MD simulations predicted that N106 inhibits NKA by interacting with the cardiotonic steroid–binding site, a well-established regulatory site of the pump. The dual modulation of SERCA2a (activation) and NKA (partial inhibition) by N106 defines a novel scaffold that enables dual regulation of cardiac ion pumps to produce positive ino-lusitropic effects in the failing heart.

## 2. Materials and Methods

### 2.1. Docking Simulations

For the docking simulations, we used CB-Dock2 [9], which combines a cavity-searching engine and AutoDock Vina 1.2 [10] for blind docking calculations. We first determined whether the CB-Dock2 molecular docking protocol accurately predicts binding modes of cardiotonic steroids co-crystallized with NKA. Through these studies, we found that the top-ranking poses predicted by CB-Dock2 predicts that cardiotonic steroids closely match the geometry of those found in the crystal structures, with RMSD values < 2 Å. Upon validation of the docking protocol, we used 28 structures of porcine NKA reported in the Protein Data Bank [11]. A list of PDB entry codes, representative states for each structure, and resolution are found in Appendix A. The 3D structure of N106 was retrieved from the PubChem database (CID: 3236395) [12], and geometry optimization and energy minimization was performed with the MMFF94s force field using the Avogadro package [13]. Blind docking of N106 was performed on 20 protein cavities predicted by the CB-Dock2 algorithm, for a total of 560 independent NKA-N106 docking calculations.

### 2.2. Molecular Dynamics Simulations

The consensus NKA-N106 complex obtained from the blind docking simulations was inserted in a in a pre-equilibrated 120 × 120 Å bilayer that mimics the composition of the plasma membrane. Specifically, the extracellular side of the membrane contained POPC and POPE (2:1 ratio) lipids, whereas the intracellular side of the bilayer was modeled using POPC, POPE, and POPS lipids (2:1:1 ratio). For lipids, we used the LIPID21 force field [14] and a cutoff distance of 10 Å for non-bonded interactions. We used the replacement method to generate lipid packing around the protein-ligand complex. We solvated each system using the OPC water model with a periodic box with a minimum margin of 20 Å between the protein and the *z*-axis edges. Na^+^, K^+^, and Cl^−^ ions were added to neutralize the system and to produce Na^+^ and K^+^ concentrations of 130 and 20 mM, respectively. The systems were prepared using the CHARMM-GUI web server [15,16]. Energy minimization and equilibration were performed as follows: two 25-ps restrained canonical ensemble (NVT) simulations, one 25-ps restrained isothermal-isobaric ensemble (NPT) simulation, and three 250-ps restrained NPT simulations, and a single unrestrained NPT simulation for 5 ns. The Langevin thermostat was used to keep the temperature at 37 °C to match that used in the ATPase assays, and the Monte Carlo barostat to maintain a constant pressure of 1.0 bar. Bonds involving hydrogen atoms were constrained using the SHAKE algorithm. We performed three independent 1-µs MD simulations of the complex using AMBER24 on Tesla V100 GPUs [17] and the AMBER ff14SB force field [18]. Data analysis was performed using VMD 2.0 [19].

### 2.3. Chemicals

All chemicals used in this study were purchased at reagent quality (purity > 95% by HPLC) and purchased from Sigma (St. Louis, MO, USA) and Thermo-Fisher Scientific (Waltham, MA, USA) unless otherwise indicated. N106 (N-(4-methoxy-1,3-benzothiazol-2-yl)-5-(4-methoxyphenyl)-1,3,4-oxadiazol-2-amine) was purchased from MedChemExpress (Monmouth Junction, NJ, USA; catalog no. HY-110273) at a purity of 99.58%.

### 2.4. NKA Isolation and Purification

NKA (α1 isoform) was prepared from porcine kidney outer medulla using the established protocols [20,21,22], with some modifications. Briefly, fresh porcine kidneys were obtained from post-mortem from the University of Michigan Unit for Laboratory Animal Medicine. The outer medulla was dissected, mixed with ice- cold buffer composed of 250 mM sucrose, 30 mM L-histidine (pH 7.3) in 1:1 (tissue:buffer) ratio, and incubated in cold-room overnight. Next day, the tissue was minced, and homogenized in ice-cold buffer containing 250 mM sucrose, 20 mM imidazole, and 1 mM EDTA (pH 7.4) using a glass-Teflon homogenizer. The homogenate was subjected to differential centrifugation at 3700× *g* for 20 min at 4 °C to followed by centrifugation of the supernatant at 7400× *g* for 20 min to remove cell debris. Subsequently, the supernatant was subjected to additional centrifugation at 38,000× *g* for 40 min to pellet the microsomal membranes. The resulting microsomal fraction was resuspended and solubilized in 0.5% (*w*/*v*) sodium dodecyl sulfate (SDS) in a buffer containing 250 mM sucrose, 0.9 mM EDTA, and 20 mM L-histidine (pH 7.0). Solubilized membranes were subjected to density-gradient ultracentrifugation. The purified enzyme was aliquoted, flash-frozen in liquid nitrogen, and stored at −80 °C in stabilization buffer containing 250 mM sucrose, 0.9 mM EDTA, and 20 mM L-histidine (pH 7.0) supplemented with a trace amount of SDS to prevent aggregation. Protein concentration was determined by the Bradford assay using bovine serum albumin as a standard, assuming a molecular weight of 165 kDa for the α/β heterodimer. Enzyme purity was verified by SDS-PAGE followed by Coomassie staining. The identity of the α1 isoform was confirmed by Western blot analysis. The enzymatic activity was verified through ouabain (OUA)-sensitive ATP hydrolysis assays prior to use in experiments.

### 2.5. ATPase Activity Assays

We measured the ATPase activity sensitive to 500 μM OUA (in μmol·min^−1^·mg^−1^) from the decrease in absorbance of NADH at 340 nm at 37 °C in a 96-well format using a Synergy H1 microplate reader (BioTek, Winooski, VT, USA). Each well contained a 200 μL final volume of assay buffer containing NKA buffer (30 mM MOPS, 130 mM NaCl, 20 mM KCl, and 4 mM MgCl2, pH = 7.4), 5U lactate dehydrogenase, 5U pyruvate dehydrogenase, 1 mM phosphoenolpyruvate, 3 mM ATP, 0.2 mM NADH, and 0.8 μg of the purified NKA suspension. Each concentration of N106 (0.1–100 μM) tested was prepared in a final volume of 200 μL and incubated with the reaction mixture for 30 min at 37 °C. To assess the ATPase activity at different concentrations of Na^+^, K^+^, or ATP and in the presence or absence of N106, we prepared serial dilutions of NaCl (0–140 mM), KCl (0–40 mM), and ATP (0–10 mM). In all cases, the residual activity in the presence of OUA was subtracted from the total estimated ATPase activity in OUA-untreated samples, and all data are presented as the OUA-sensitive ATPase activity. IC_50_ values were calculated from the fitted concentration-response curve using GraphPad Prism 10 (Boston, MA, USA). Data are reported as mean ± SEM (*n* = 3–4).

## 3. Results

### 3.1. Docking Studies Reveal That N106 Interacts with the Cardiotonic Steroid-Binding Site of NKA

**Figure 1:**
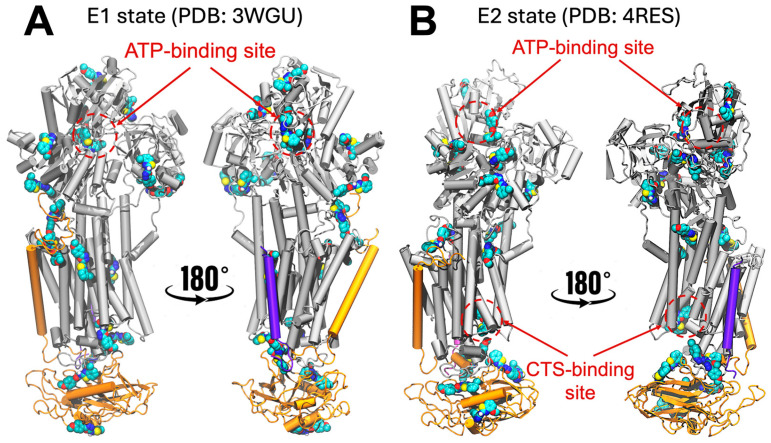
NKA–N106 interactions predicted by the combined cavity detection and blind docking simulations. The structures represent the results of extensive docking simulations using a combined 28 crystal structures of NKA representing the (**A**) E1 state (represented here by the PDB entry 3WGU [25]) and (**B**) the E2 state (represented here by the PDB entry 4RES [26]). We show the location of the nucleotide- and CTS-binding sites are shown for clarity. N106 bound at different sites of the protein, as predicted by the docking simulations, are shown as van der Waals spheres. The protein structure is shown as cartoons, highlighting the α-subunit (gray), β-subunit (orange) and γ-subunit (purple) of NKA.

To identify potential interactions between NKA and N106, we first performed blind docking using 28 structures of porcine NKA available in the Protein Data Bank (PDB). Collectively, these 28 structures encompass the two major conformational states of the pump, namely the E1 (high affinity for Na^+^) and E2 (high affinity for K^+^) states of the pump. We found that the docking simulations predict N106 binding to several pockets in the E1 state of NKA, all of which are located either in the intracellular or extracellular domains of the protein. Representative docking poses are shown in Figure 1A. However, most of the predicted sites have no functional significance as they have not been validated either structurally or biochemically using small molecules, including interactions with the cytosolic and transmembrane domains. N106 is predicted to interact with functionally relevant sites, including near the nucleotide-binding site and near the extracellular segment of the transmembrane helix M4, which is critical for opening the ion pathway to the extracellular space [23]. However, in the E1 state, stabilization of N106 is primarily mediated by the autophosphorylation site which interacts with the oxadiazole moiety of the compound, while no other significant intermolecular contacts appear to support binding at this site (Figure 1A). Likewise, N106 interacts with NKA near the extracellular domain, but no additional intermolecular contacts appear to stabilize this interaction (Figure 1A).

Docking of N106 to the E2 state showed mostly non-specific interactions with either the intracellular domain of NKA or the extracellular domain of the β-chain of NKA; a representative location of the binding poses is shown in Figure 1B. As with the E1 state, we found N106 interacting near the nucleotide-binding site, albeit through non-specific molecular interactions (Figure 1B). Interestingly, the docking predictions indicate that N106 engages the canonical cardiotonic steroid (CTS)–binding site (Figure 1B), which serves as the main regulatory pocket that allosterically stabilizes the phosphorylated E2 conformation of the pump and thereby mediates inhibition of NKA activity. This binding mode mirrors that of classical CTSs, suggesting that N106 may exert its functional effects by occupying the same structural cavity that controls the conformational equilibrium between E1 and E2 states. Occupation of this pocket is known to restrict the large-scale domain rearrangements required for ion transport, ultimately locking the pump in an E2-P–like state with reduced turnover [24]. The docking scoring function indicates that N106 binds to the CTS-binding site with docking scores ranging from about −8 to −9 kcal/mol. We note that while these values do not represent the actual free energy of binding, they indicate that N106 interacts favorably with the CTS-binding site.

### 3.2. N106 Is a Partial Inhibitor of NKA Without Altering Its Affinity for K^+^/Na^+^ or ATP

**Figure 2:**
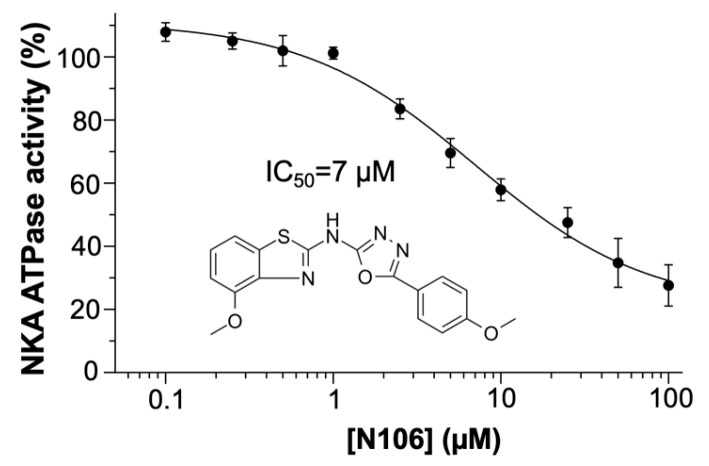
Ten-point concentration-response curve of N106 against NKA. The activity of the compounds at each concentration represents the OUA-sensitive ATPase activity obtained at 130 mM NaCl and 20 mM KCl and normalized relative to the untreated control as described in Experimental procedures. The 2D structure of N106 is shown within the plot. Data are reported as average ± SEM (*n* = 4).

We performed ATPase assays to investigate whether N106 inhibits NKA activity. To this aim, we measured OUA-sensitive, specific ATPase activity using purified enzyme preparations. In all cases, activity is represented as the % of the change in specific activity relative to the activity of untreated NKA (negative control). When describing the data, we make a distinction between ‘observed’ data (i.e., datapoints) and ‘estimated’ data (i.e., values derived from the fitted model data). We found that N106 produced a concentration-dependent decrease in ATPase activity, with an estimated IC_50_ of 7 ± 1 μM (*n* = 4; Figure 2), in agreement with our docking predictions. We observed that enzymatic activity plateaued at approximately 20–30% of the control value at the highest concentration tested here (i.e., 100 µM). Furthermore, the fitted model indicates that the maximal estimated inhibition is ~80% at compound concentrations > 100 µM, indicating that N106 is a partial inhibitor of the pump. This incomplete inhibition suggests that the compound partially stabilizes catalytically competent conformations of NKA, rather than fully blocking turnover as seen with classical CTS.

**Figure 3:**
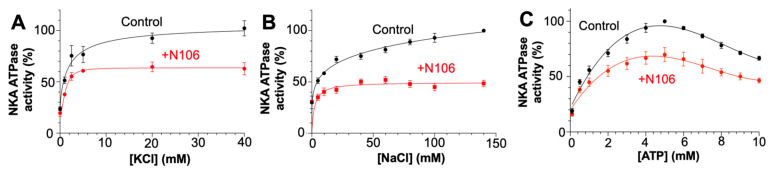
Effect of N106 on NKA activity under varying Na^+^, K^+^ and ATP concentrations. NKA was assayed for ATPase activity in the absence (black) or presence (red) of 10 µM N106. We evaluated the changes in NKA activity at increasing concentrations of (**A**) KCl and (**B**) NaCl. (**C**) shows the changes in NKA activity at increasing ATP concentrations. Data are expressed as mean ± SEM (*n* = 3–4).

To further investigate the mechanism underlying inhibition of NKA by N106, we examined how this compound affects substrate and K^+^- or Na^+^-dependent activation of NKA. In assays varying KCl concentration (Figure 3A), control samples showed typical hyperbolic activation of ATPase activity, reaching maximal stimulation near 20–30 mM of KCl. In the presence of N106, the activity was reduced by approximately 50%, without changes in apparent affinity for K^+^. A similar trend was observed at various NaCl concentrations (Figure 3B), where N106 lowered the maximal rate of [NaCl]-dependent activation but had only minor effects on apparent Na^+^ affinity. These results indicate that N106 predominantly affects the enzyme’s catalytic efficiency rather than its ion-binding properties. Furthermore, the reduction in activity without major changes in cation affinity suggests that N106 perturbs the conformational transitions coupling Na^+^ and K^+^ binding to ATP hydrolysis, likely by stabilizing an E2-like state with reduced turnover, in agreement with the docking predictions indicating that N106 binds to the CTS-binding site.

We next evaluated whether N106 alters ATP-dependent activation of NKA. Under control conditions, enzyme activity increased with ATP concentration, reaching maximal specific activity at ATP concentrations of 4–5 mM (Figure 3C). The presence of N106 decreased the maximal ATPase rate by roughly 40–50%, yet the apparent ATP affinity remained unchanged. These findings are consistent with a noncompetitive inhibitory mechanism, in which N106 reduces catalytic turnover without interfering directly with ATP binding. This kinetic profile indicates that N106 inhibits the ATPase activity of NKA without competing with ATP for binding, in agreement with the docking simulations.

### 3.3. Molecular Dynamics Simulations of the NKA-N106 Complex Provide an Explanation for the Partial Inhibition Observed in ATPase Assays

The ATPase assays indicate that N106 inhibits NKA, but the inhibitory pattern of N106 differs from that of classical CTS ligands. Specifically, OUA and related steroids fully inhibit NKA by stabilizing the phosphorylated E2-P state, preventing ion release and turnover. In contrast, N106 only partially suppresses enzymatic activity, suggesting a distinct binding mode or a less restrictive stabilization of E2-like conformations. Yet, molecular docking and simulation analyses indicate that N106 occupies the CTS pocket and interacts with residues that stabilize CTS. It is possible that the docking simulations do not fully capture the extent of the interaction between N106 and the CTS-binding site, which may result in incomplete occlusion of the cation-binding sites, allowing residual cycling and ATPase activity. Such a mechanism may explain both the partial inhibition and the preserved responsiveness to ion and ATP substrates observed experimentally. To test this mechanism, we performed unbiased, 1-μs MD simulations of the NKA–N106 complex and analyzed the frequency of residue–ligand contacts.

**Figure 4:**
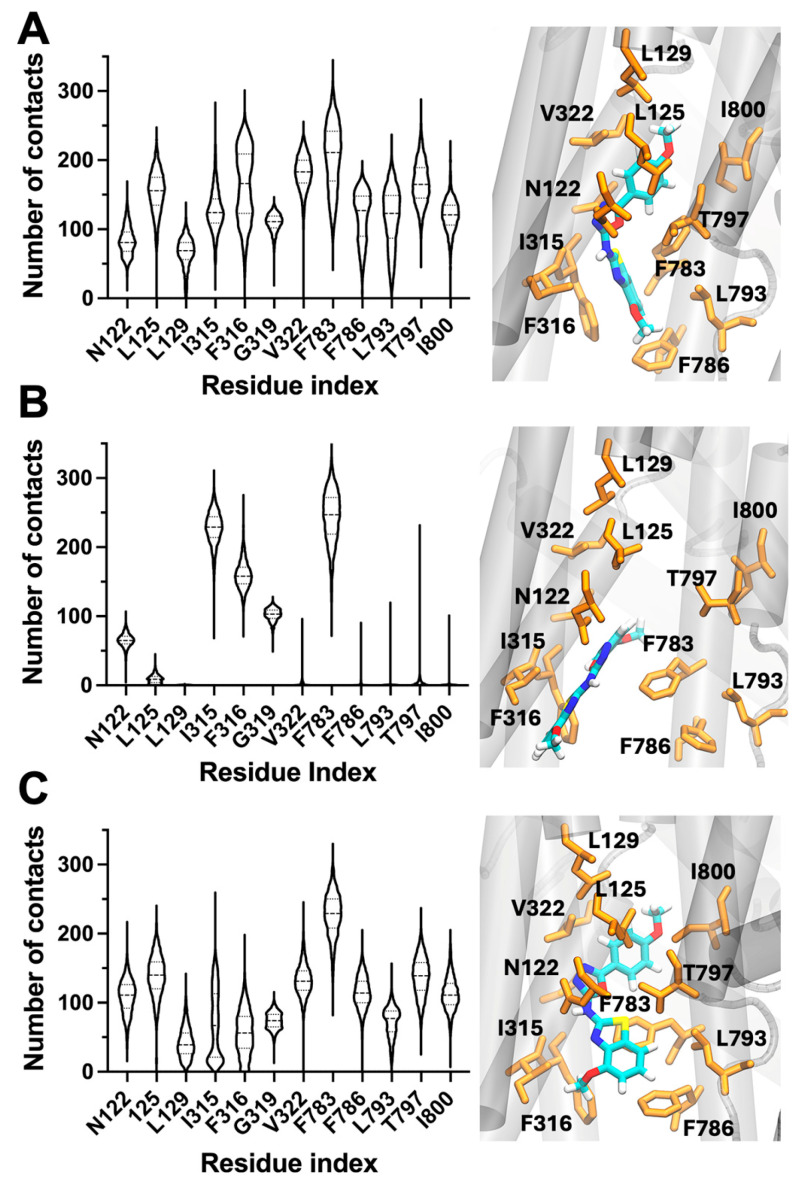
Dynamic binding behavior of N106 within the CTS-binding sites of NKA revealed by MD simulations. Panels (**A**–**C**) correspond to three independent 1-μs MD replicas. Violin plots depict residue–ligand contact frequencies, and accompanying snapshots illustrate representative binding poses. Dashed lines represent quartiles and the median, and widths represent the number of structures with the same number of contacts. Across simulations, N106 consistently interacts with hydrophobic residues L125, L129, I315, F316, and V322, anchoring the ligand within the cardiotonic-steroid binding site. Variability in contacts with F783, F786, and T797 interface reflects partial disengagement of the cytoplasmic region, consistent with a dynamically stabilized, partially inhibitory binding mode. In all cases, NKA is shown as gray ribbons, and key NKA residues orange sticks. N106 is shown as sticks and colored by atom type.

Analysis of the MD trajectories revealed that N106 remained stably accommodated within the transmembrane cavity that corresponds to the canonical CTS binding region. The ligand generally maintained persistent interactions with a hydrophobic cluster composed of L125, L129, I315, F316, and V322, which anchored the benzothiazolamine and linker moieties deep within the pocket. These contacts dominated across all simulations, underscoring a well-defined hydrophobic core that stabilizes N106 in the inhibitory site (Figure 4). Notably, the simulations also revealed variability in contacts near the cytoplasmic end of the pocket. In simulation A, N106 formed frequent and sustained interactions with residues F783, F786, and T797, suggesting a compact binding pose that bridges the upper and lower segments of the cavity (Figure 4A). In contrast, simulation B showed weakening of these interactions, with the compound retaining the core hydrophobic contacts but detaching from the canonical binding site. This behavior was accompanied by transient exposure of the terminal amide group to the lipid phase, indicating increased flexibility and partial unbinding of the cytoplasmic tail (Figure 4B). Simulation C reproduced a similar pattern to simulation A, although the contacts with F786 and T797 were less stable, again pointing to a dynamic equilibrium between bound and semi-unbound conformations (Figure 4C). Together, the simulations predict that N106 does not fully immobilize within the CTS pocket but instead samples multiple binding substates that differ in their engagement of residues at the CTS-binding pocket. Therefore, the intermittent formation and loss of contacts provide a plausible structural hypothesis for the partial inhibitory profile of N106 observed in ATPase assays and highlights the importance of maintaining stable interactions for complete inhibition of catalytic activity.

## 4. Discussion

In this study, we used molecular simulation and ATPase assays to investigate whether N106, an activator of cardiac SERCA2a through stimulation of SUMOylation [7], inhibits NKA. Biochemical assays demonstrate functional inhibition of NKA, showing that N106 partially (~80%) inhibits the pump in a concentration-dependent manner. The ATPase assays also showed that the compound reduces maximal ATPase activity without altering the apparent affinities for Na^+^, K^+^, or ATP, consistent with a noncompetitive mechanism. This behavior suggests that N106 perturbs conformational transitions coupling ion binding to phosphorylation and dephosphorylation rather than directly competing for substrate binding sites.

Complementary docking and MD simulations predicted the canonical cardiotonic-steroid (CTS) binding pocket as the primary site of interaction. These simulations suggest that N106 remains anchored within the transmembrane cavity by a hydrophobic core of residues (L125, L129, I315, F316, and V322) shared with the CTS-binding pocket, but the ligand exhibits some mobility in this binding site. In particular, the intermittent loss of contacts with residues F783, F786, and T797 allows transient partial unbinding of the terminal moiety toward the lipid phase. These predictions suggest that N106 stabilizes an E2-like conformation of the enzyme that slows but does not completely block ATPase activity. Therefore, we propose that this dynamic binding behavior likely underlies the residual enzymatic activity observed experimentally. Future studies, such as competitive displacement assays using a labeled CTS (e.g., ^3^H-ouabain) or direct binding assays employing labeled N106, will be required to test this structural hypothesis.

From a pharmacological standpoint, the resulting partial inhibition may have important physiological implications, as it could modulate intracellular Na^+^ levels and Ca^2+^ handling characteristic of HF [27] while mitigating some of the cardiac toxicity effects exerted by CTS [28,29]. Indeed, full inhibition of NKA is well known to produce narrow therapeutic windows and arrhythmogenic risk [28,29], whereas moderate suppression can increase contractility through moderate elevation of intracellular Na^+^ while activation of cardiac SERCA2a through SUMOylation enhances Ca^2+^ reuptake into the sarcoplasmic reticulum, promoting efficient relaxation and restoring systolic–diastolic balance. This is important because cell-based assays have shown that the highest effects on contractility and relaxation are achieved at a concentration of 10 µM [7], which falls within the concentration at which N106 inhibits NKA in our assays. Together, these complementary effects could explain the compound’s balanced ino-lusitropic properties that have been observed for N106 in vitro [7]. Overall, N106 offers a promising lead for developing optimized derivatives that fine-tune cardiac contractility while minimizing proarrhythmic risk, representing a new paradigm for HF therapy.

While this study suggests that NKA inhibition underlies the positive inotropic effects of N106, it remains possible that additional inotropic mechanisms contribute to its observed pharmacological profile [7]. Indeed, pathways such as myosin activation (e.g., by Omecamtiv Mecarbil [30,31,32]), calcium sensitization (e.g., by Levosimendan [33,34]), or phosphodiesterase-3 inhibition (e.g., by Milrinone [35,36]) may also be affected directly or indirectly by N106. Future studies should assess whether N106 influences these complementary mechanisms of contractility modulation. We also note that while the compound exhibits ino-lusitropic effects in cardiac cells, its reported maximal plasma concentration (C_max_ ≈ 2.2 μM [7]) falls slightly below the IC_50_ values we determined for NKA inhibition. Therefore, structural optimization aimed at enhancing potency could enable more effective translation of N106’s dual modulatory effects.

## 5. Conclusions

N106 partially inhibits without altering the pump’s apparent affinity for Na^+^, K^+^, or ATP. Docking analyses and microsecond-scale MD simulations predict that N106 occupies the CTS-binding site but undergoes transient unbinding events, a dynamic behavior consistent with the partial inhibition observed experimentally. Together, these findings support the concept that N106 functions as a first-in-class dual modulator of cardiac ion pumps, combining partial NKA inhibition (this work) with previously reported stimulation of SERCA2a activity [7].

## Data Availability

Data is contained within the article.

## Abbreviations

The following abbreviations are used in this manuscript:

ATP	Adenosine triphosphate
CTS	Cardiotonic steroids
HF	Heart failure
IC_50_	Half-maximal inhibitory concentration
MD	Molecular dynamics
NKA	Na^+^/K^+^-ATPase
OUA	Ouabain
PDB	Protein Data Bank
SERCA2a	Sarcoplasmic/endoplasmic reticulum Ca^2+^-ATPase isoform 2a
SUMO	Small ubiquitin-like modifier

## Appendix A

**Table A1:** List the accession codes, assigned conformational states, and resolutions of the 28 porcine NKA structures used for the docking studies.

PDB Accession Code	Assigned State	Resolution
3B8E	E_2_P_i_	3.5 Å
3KDP	E_2_P_i_	3.5 Å
3N23	E_2_P	4.6 Å
4HYT	E_2_P	3.4 Å
4RES	E_2_P-2K^+^	3.4 Å
4RET	E_2_P-Mg	4.0 Å
4XE5	E_2_P	3.9 Å
6KPU	E_2_P-Mg	4.62 Å
6KPV	E_2_P-Mg	3.72 Å
6KPX	E_2_P-Mg	3.72 Å
6KPW	E_2_P-Mg	3.46 Å
6KPZ	E_2_P-Mg	3.00 Å
6KQ0	E_2_P-Mg	3.50 Å
6KPY	E_2_P-Mg	3.71 Å
7D91	E_2_P-Mg	3.35 Å
7D92	E_2_P-Mg	3.90 Å
7D93	E_2_P-Mg	3.65 Å
7D94	E_2_P-Mg	3.50 Å
7DDJ	E_2_P-Mg	2.90 Å
7DDI	E_2_P-Mg	3.72 Å
7DDH	E_2_P-Mg	3.46 Å
7DDL	E_2_P-Mg	3.20 Å
7DDK	E_2_P-Mg	3.50 Å
7WYS	E_2_P-Mg	3.71 Å
7WYT	E_2_P-Mg	2.90 Å
4HQJ	E_1_P_i_-ADP	4.3 Å
3WGU	E_1_P_i_-ADP	2.8 Å
3WGV	E_1_P_i_-ADP	2.8 Å
